# What’s Wrong with Human/Nonhuman Chimera Research?

**DOI:** 10.1371/journal.pbio.1002535

**Published:** 2016-08-30

**Authors:** Insoo Hyun

**Affiliations:** Department of Bioethics, Case Western Reserve University School of Medicine, Cleveland, Ohio, United States of America

## Abstract

The National Institutes of Health (NIH) is poised to lift its funding moratorium on research involving chimeric human/nonhuman embryos, pending further consideration by an NIH steering committee. The kinds of ethical concerns that seem to underlie this research and chimera research more generally can be adequately addressed.

## Abstract 2

This Perspective argues that the ethical concerns behind the NIH funding moratorium on chimeric embryo research can be adequately and reasonably addressed.

Stem-cell–based human/nonhuman chimera research involves the transfer of human stem cells into animal hosts at various stages of development. The purpose of this research is to introduce localized human cellular and biological characteristics into laboratory animals to advance stem cell science, developmental biology, and many areas of biomedicine. Human/nonhuman chimera research has existed without much controversy for decades outside of stem cell research, resulting in, for example, mouse models of human cancer and the human immune system [1]. However, the possibility of acute levels of human/nonhuman mixing in stem-cell–based chimeras seems to be of special concern to many, as I discuss in this Perspective piece.

The debate over stem-cell–based chimeras is no mere philosophical quibble: it has real, practical import for research funding. Chimera research was thrust into the spotlight in September 2015 when the National Institutes of Health (NIH) announced a moratorium on the funding of research in which human pluripotent stem cells are transferred into nonhuman vertebrate pre-gastrulation stage embryos (http://grants.nih.gov/grants/guide/notice-files/NOT-OD-15-158.html). Now the NIH has announced it might allow public funds for this type of research under a proposed policy that would utilize input from a steering committee established to assess and oversee human/nonhuman chimera research (https://federalregister.gov/a/2016-18601). Until this new approach is finalized, however, the current moratorium will remain in place.

Interestingly, the NIH moratorium does not include other forms of human/nonhuman mixing that would seem to be in the same ethical ballpark as human stem cell transfers into animal embryos, such as genetic humanizations of mice [2] and human glial progenitor cell transplantations into neonatal mouse brains [3]. One may wonder why this is the case.

The NIH’s funding moratorium appears to have been triggered by a branch of stem cell research that aims to grow transplantable human organs in genetically modified large animals, such as pigs and sheep. Researchers have shown that it is possible to grow a rat pancreas in a mouse and vice versa by “complementing” the preimplantation embryo (blastocyst) of one species with pluripotent stem cells of the other once the embryo has been genetically modified in vitro to lack its own pancreas [4]. Building on the success of these techniques, researchers are now interested in complementing pancreatogenesis-disabled pig embryos with primitive, so-called “naïve” human pluripotent stem cells for a similar purpose: to grow a human pancreas in a pig [5]. This line of research, which is currently being supported with nonfederal funds, could open the door to growing various types of human organs such as hearts and kidneys in livestock animals for transplantation in the future. If the pluripotent stem cells for transfer are created using a patient’s own tissue sample, then, in theory, the resulting organ would be immune-compatible with the patient. The humanitarian importance of this research is both apparent and urgent. There is currently a dire shortage of organs for transplantation in the United States, leading to approximately 22 deaths per day among patients waiting for organs.

Given the noble aims of this research, it is puzzling to some why the NIH is so nervous about providing federal funds to researchers with a track record of success in this area. The NIH has for years supported research in which human cells are transplanted into animal models, and it continues to fund human/nonhuman chimera research that lies outside the scope of research singled out in its notice of moratorium. How might this current policy difference be explained?

One possible answer is that there may be unique ethical risks involved in growing human organs in animals. In particular, some might worry that embryo complementation could inadvertently affect the developing animal in ways that go well beyond the localized formation of human organs, resulting in an acutely chimeric animal with an ambiguous moral status. Apparently, the threat of such an outcome simply may not be held to be as present in non-stem-cell–based chimera research. The transfer of cancerous human cells into postnatal immune-deficient rodents or the swapping of a mouse’s immune system with a human one arouses little ethical concern because these forms of chimerism are limited to more mature cell types and anatomical sites that have no obvious bearing on an animal’s moral status.

Unlike these other examples of human/nonhuman chimerism, stem-cell–based chimerism has the potential to radically humanize the biology of laboratory animals, depending on the type and number of human stem cells transplanted, the species and developmental stage of the host animal, and the anatomical location of the animal host where the human stem cells are transferred [6]. When human stem cells are transplanted into a postnatal animal, it is unlikely these cells will integrate significantly into the animal’s existing biological structures. But if human stem cells are introduced into an embryonic or fetal animal host that is then gestated, then the fractional percentage of differentiating human cells and the degree of human physiological integration in the developing chimeric animal may turn out to be high, especially if there is little evolutionary distance between the animal species used and humans. The worry, therefore, is that in the process of biologically humanizing a research animal, scientists may end up also *morally humanizing* the resulting chimera, especially if there is acute human/nonhuman chimerism of the central nervous system.

It is easy, however, to overstate the concern about the moral humanization of acute human/nonhuman chimeras, and several considerations should serve to turn down the heat around this speculative concern. First, the interspecies boundary that exists between humans and livestock is sufficiently high that it is quite unlikely that acute chimerization of all aspects of the resulting animal will occur. As a case in point, the mouse/rat chimeric experiments produced about 20% donor chimerism on average outside the relevant organ niche. The interspecies barrier is far lower between these rodents than it is between humans and livestock, thus making the degree of possible “off-target” human chimerism much less worrisome in the host animal. (Indeed, the evolutionary distance between pig and human is actually greater than the distance between human and mouse.) Second, in order to ensure even further that livestock blastocyst complementation using human stem cells avoids unwanted chimerism of the animal, especially within the central nervous system, researchers are now developing what they call “targeted organ generation” [7]. Here, the transferred human stem cells would be genetically modified to differentiate down only the endodermal lineage that produces the organs of interest, thus preventing the possibility that they would develop into human neural cells, which are derived from the ectodermal lineage. And third, researchers can take care to proceed in a stepwise fashion through a series of pilot studies, stopping their chimera experiments each time well before the full gestation cycle to examine fetal tissues for any unwanted migration and development of human cells outside the organ niche environment.

The precautionary steps outlined above are consistent with the ethical standards for chimera research set forth by the Ethics and Public Policy Committee of the International Society for Stem Cell Research (ISSCR), which was offered as a resource for the global stem cell community in 2007 [8]. This advisory report builds its recommendations on top of animal welfare principles that are already in play for the use of genetically modified animals, which include: (1) the establishment of baseline animal data; (2) ongoing data collection during research concerning any deviation from the norms of species-typical animals; (3) the use of small pilot studies to ascertain any welfare changes in modified animals; and (4) ongoing monitoring and reporting to oversight committees. In addition to these four conditions, the ISSCR Ethics Committee recommends that ethical assessments of chimera research should be based on rational, practical, fact-based assessments of the developmental trajectories that are likely to be affected, taking into account the biological context in which the human cells are going to be deployed. More attention needs to be placed on animal welfare standards, especially as they pertain to livestock animals for research, and less focus should be placed on speculative concerns about the “moral humanization” of chimeric animals. I conclude with a few words on this last point.

If by “moral humanization” one means the appearance of uniquely human psychological characteristics in a chimeric animal, then two considerations expose the unrealistic assumptions undergirding this concern. The first consideration is that it is entirely unclear what types of new psychological characteristics could count to elevate the moral status of a research animal above where it currently is, such that its scientific use would no longer be morally acceptable. In my view, the only characteristic that might qualify doing this heavy moral lifting is the appearance of human-like self-consciousness, defined as an existential awareness and concern for oneself as a temporally extended agent with higher-order beliefs about one’s own mental experiences. But this unique psychological characteristic is not likely to emerge in a chimeric animal’s brain, as it takes several years to develop in infant brains that are 100% human and only under the right social and nurturing conditions of child-rearing [9]. The second consideration is that people tend to assume the presence of human cells in an animal’s brain might enhance it above its typical species functioning. Yet this seems to be an extreme form of anthropocentric arrogance—an unstated moral imperialism connected to human stem cells—to assume that the presence of human neural matter in an otherwise nonhuman brain will end up improving the animal’s moral and cognitive status. The much more likely outcome of neurological chimerism is not moral humanization of the animal in this sense but rather an increased chance of animal suffering and acute biological dysfunction and disequilibrium, if our experience with transgenic animals can be a guide. This is why the ethics and regulation of chimera research should prioritize animal welfare principles while at the same time enabling scientific progress in areas of humanitarian importance, albeit in a manner consistent with these principles.

In this Perspective piece, I have attempted to lay bare the sort of concerns that appear to underlie the NIH’s moratorium on chimera research funding. I suggest that most, if not all, of these concerns can be reasonably addressed. Although stem-cell–based chimera research encompasses a wide array of research activities, the issues surrounding the NIH moratorium nicely encapsulate the ethical concerns that are common across many forms of chimera research. Thus, my analysis in this Perspective might provide an efficient way to target the ethics of chimera research more generally.

## Abbreviations

ISSCR: International Society for Stem Cell Research
NIH: National Institutes of Health
